# Electrophysiological approach to determine kinetic parameters of sucrose uptake by single sieve elements or phloem parenchyma cells in intact *Vicia faba* plants

**DOI:** 10.3389/fpls.2013.00274

**Published:** 2013-07-31

**Authors:** Jens B. Hafke, Sabina-Roxana Höll, Christina Kühn, Aart J. E. van Bel

**Affiliations:** ^1^Institute of Plant Physiology, Justus-Liebig-UniversityGiessen, Germany; ^2^Institute of General Botany, Plant Cell Biology Research Group, Justus-Liebig-UniversityGiessen, Germany; ^3^Department of Plant Physiology, Institute of Biology, Humboldt UniversityBerlin, Germany

**Keywords:** Akaike's Information Criterion, carbohydrate partitioning, Eadie–Hofstee plots, H^+^/sucrose symporter, *K*_*m*_ values, non-linear least-square fittings, phloem parenchyma cells sieve-element/companion cell complex, sucrose-induced depolarization of membrane potential

## Abstract

Apart from cut aphid stylets in combination with electrophysiology, no attempts have been made thus far to measure *in vivo* sucrose-uptake properties of sieve elements. We investigated the kinetics of sucrose uptake by single sieve elements and phloem parenchyma cells in *Vicia faba* plants. To this end, microelectrodes were inserted into free-lying phloem cells in the main vein of the youngest fully-expanded leaf, half-way along the stem, in the transition zone between the autotrophic and heterotrophic part of the stem, and in the root axis. A top-to-bottom membrane potential gradient of sieve elements was observed along the stem (−130 mV to −110 mV), while the membrane potential of the phloem parenchyma cells was stable (approx. −100 mV). In roots, the membrane potential of sieve elements dropped abruptly to −55 mV. Bathing solutions having various sucrose concentrations were administered and sucrose/H^+^-induced depolarizations were recorded. Data analysis by non-linear least-square data fittings as well as by linear Eadie–Hofstee (EH) -transformations pointed at biphasic Michaelis–Menten kinetics (2 MM, EH: *K*_*m*1_ 1.2–1.8 mM, *K*_*m*2_ 6.6–9.0 mM) of sucrose uptake by sieve elements. However, Akaike's Information Criterion (AIC) favored single MM kinetics. Using single MM as the best-fitting model, *K*_*m*_ values for sucrose uptake by sieve elements decreased along the plant axis from 1 to 7 mM. For phloem parenchyma cells, higher *K*_*m*_ values (EH: *K*_*m*1_ 10 mM, *K*_*m*2_ 70 mM) as compared to sieve elements were found. In preliminary patch-clamp experiments with sieve-element protoplasts, small sucrose-coupled proton currents (−0.1 to −0.3 pA/pF) were detected in the whole-cell mode. In conclusion (a) *K*_*m*_ values for sucrose uptake measured by electrophysiology are similar to those obtained with heterologous systems, (b) electrophysiology provides a useful tool for *in situ* determination of *K*_*m*_ values, (c) As yet, it remains unclear if one or two uptake systems are involved in sucrose uptake by sieve elements, (d) Affinity for sucrose uptake by sieve elements exceeds by far that by phloem parenchyma cells, (e) Patch-clamp studies provide a feasible basis for quantification of sucrose uptake by single cells. The consequences of the findings for whole-plant carbohydrate partitioning are discussed.

## Introduction

The wide-spread notion that carbohydrate distribution largely results from competition between terminal sinks neglects the role of axial sinks along the pathway and the dynamics of phloem transport (van Bel, 1996, 2003a; Hafke et al., 2005). Studies using ^11^C-labeled substances revealed considerable rates of photoassimilate exchange along the axis of intact *Phaseolus* plants (Minchin and Thorpe, 1984, 1987). The results indicate a dynamic leakage/retrieval of photoassimilates along the transport phloem (Eschrich et al., 1972; Minchin and Thorpe, 1987; van Bel, 2003b; Gould et al., 2012). Under mostly prevailing source-limiting conditions when sink demand exceeds source supply (Patrick and Offler, 1996), photoassimilates translocated in the sieve-tube sap are steadily leaking into the vascular apoplasmic space. From there, photoassimilates are retrieved by sieve-element/companion-cell complexes (SE/CCs) or absorbed by phloem parenchyma cells (PPCs), which present the border-line of the axial sink area (van Bel, 1996). Competition between SE/CCs and PPCs for photoassimilates was postulated to be a key process regulating photoassimilate partitioning between axial and terminal sinks (van Bel, 1996; Hafke et al., 2005; van Bel and Hafke, 2005). The balance between release and retrieval may also be involved in the maintenance of the hydraulic pressure gradient in transport phloem (Minchin and Thorpe, 1987; van Bel, 1996; Gould et al., 2004; van Bel and Hafke, 2005).

Under source-limiting conditions, SE/CCs and PPCs seem to represent syplasmically separated domains in transport phloem (van der Schoot and van Bel, 1989; Oparka et al., 1994; van Bel and van Rijen, 1994; Patrick and Offler, 1996; Rhodes et al., 1996; Hafke et al., 2005). Hence, photoassimilate uptake by either SE/CCs or PPCs is dictated by the respective proton motive forces (pmf; e.g., van Bel, 1996). The competitiveness between SE/CCs and PPCs was quantified by pmf measurements in the main vein of intact *Vicia faba* plants and a few other plant species (Hafke et al., 2005). These studies identified the membrane resting potential as the domineering pmf-component in the competition between SE/CCs or PPCs.

Apart from the pmf, other parameters, in particular *K*_*m*_ and *V*_max_ of the sucrose transporters determine the competitiveness for photoassimilates between SE/CCs and PPCs. To date, sucrose-uptake kinetics of sieve elements has been determined by using entire phloem tissues or sucrose transporters expressed in oocytes and yeast (for reviews see Kühn, 2003; Lalonde et al., 2003; Sauer, 2007; Ayre, 2011; Geiger, 2011). These approaches do not provide conclusive information on the *in situ* uptake by sieve elements. In the first approach, uptake kinetics of sieve elements are blurred by the contribution of other cell types; in heterologous expression, uptake is taking place in an artificial environment and fails to quantify *in situ V*_max_ of sucrose uptake. Furthermore, kinetics of sucrose uptake by PPCs has been largely neglected so that the picture of the competition between SE/CCs and PPCs is far from complete.

The lack of exact information calls for approaches by which the sucrose-uptake kinetics of each single cell type can be measured separately in the natural cell environment. Therefore, we executed depolarization studies (cf. Lichtner and Spanswick, 1981; Wright and Fisher, 1981) using intracellular electrodes. They were impaled into phloem cells to record electrical responses to graded sucrose supply. The magnitude of depolarization allows the calculation of the *K*_*m*_ of the transporters involved, but did not provide information on *V*_max_. The *V*_max_ values depend on the transporter density and the turnover rates. Hence, the feasibility of a whole-cell patch-clamp approach was explored to detect and possibly quantify sucrose uptake by sieve-element protoplasts.

## Methods

### Plant material

*Vicia faba* cv. Witkiem plants (Nunhems Zaden BV, Haelen, The Netherlands) were grown in pots in a greenhouse at temperatures varying between 20°C and 30°C at 60–70% humidity and a 14/10-h light/dark period. Supplementary lamp light (model SONT Agro 400 W; Phillips Eindhoven, The Netherlands) resulted in an irradiance level of 200–250 μmol m^−2^ s^−1^ at the plant apex. Test plants were taken 3 weeks after germination (cf. Hafke et al., 2005).

#### Tissue preparation of intact plants

Cortical layers of the main vein of the youngest mature leaf or of internodal rims were removed by manual paradermal slicing with a fresh razor blade as described before (Knoblauch and van Bel, 1998). Leaves were mounted on a microscope slide with two-sided adhesive tape and the free-lying tissue was bathed in a weakly buffered standard bathing medium (BM): 2 mM KCl, 1 mM CaCl_2_ 1 mM MgCl_2_, 100 mM mannitol, 2.5 mM 4-morpholinoethanesulfonic acid (MES)/NaOH, pH 5.7. For measurements in internodes being bent into a horizontal plane, a home-made bathing system was used to submerse and perfuse free-lying phloem tissue with various solutes. Intactness of phloem tissue was checked using a microscope (Leica DM-LB, fluorescence microscope) equipped with a water immersion objective (HCX APO L40x/0.80 W U-V-I objective, Leica, Heidelberg, Germany) that was insulated from the electrophysiological devices.

#### Intracellular electrophysiology in intact plants

**Figure 1:**
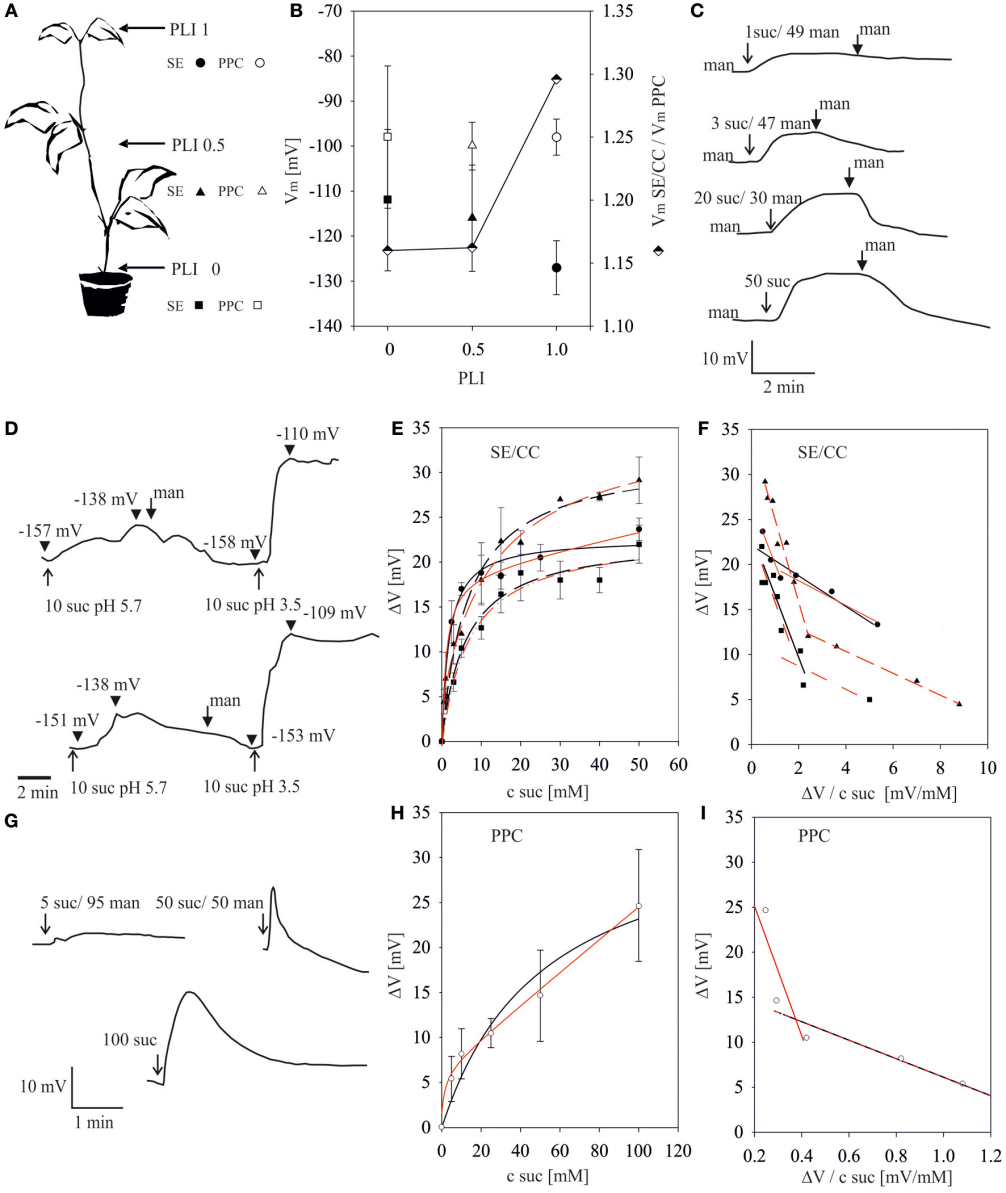
**Sucrose-induced depolarizations in sieve-elements (SEs) and phloem parenchyma cells (PPCs) along the stem of intact *Vicia faba* plants**. **(A)** Plant length indices (PLIs: 1.0, mid-vein of the youngest mature leaf; 0.5, half-way the stem; 0, stem-to-root transition area) standardize the microelectrode positions along plants of diverse lengths. The symbols (circles, triangles, squares) associated with certain PLIs are used in the following figures. The numbers associated with sucrose (suc) and mannitol (man) in the following figures represent their concentrations (mM). **(B)** Membrane potential resting levels (*V*_m_, ±SD, left *y*-axis) in SE/CCs and PPCs along the plant axis. Membrane potential ratios (*V*_mSE/CC_/*V*_mPPC_, semi-filled diamonds, right *y*-axis) along the phloem pathway. **(C)** Typical recorder traces showing the time-course of the change in SE membrane potentials at PLI 1.0 in response to the addition of 1, 3, 20 and 50 mM sucrose, respectively. The start of perfusion with test solutions is marked by arrows, that of mannitol rinsing by fat arrows. **(D)** pH-dependence of sucrose uptake into single SEs. Depolarizations in response to 10 mM sucrose pH 5.7 and 10 mM sucrose pH 3.5 intermitted by rinsing with mannitol. The start of perfusion with test solutions is marked by arrows, that of mannitol rinsing by fat arrows. Arrowheads mark the membrane voltage. **(E)** Relationship between sucrose-induced depolarizations (*y*-axis) of SEs and external sucrose concentrations at pH 5.7 (*x*-axis) at different PLIs (*n* = 5 to 10 for each concentration). Data points where fitted either to a single MM term (black line) or to the biphasic MM equation (red line) **(F)** Eadie–Hofstee transformation of sucrose-induced depolarization as a measure for sucrose uptake at different PLIs for SEs. In Eadie–Hofstee plots, the negative slopes of linear fits represent the -*K*_*m*_ values. For linear regressions of the data from PLI 1 and PLI 0 either a single MM (black line) or a biphasic MM (red line) kinetic is assumed. For PLI 0.5 two components were unequivocally identified **(G)** Typical recorder traces showing the time-course of the change in PPC membrane potential at PLI 1.0 in response to 5, 50 and 100 mM sucrose, respectively. **(H)** Relationship between membrane potential depolarizations (ΔV, *y*-axis) and supplied sucrose concentrations (*x*-axis) of PPCs at PLI 1 at pH 5.7 (*n* = 10 for each concentration). Data points where fitted either to a single MM term (black line) or biphasic MM term (red line). **(I)** Eadie–Hofstee transformation of sucrose-induced depolarization of PPCs at PLI 1.

Membrane potential measurements in intact phloem tissue have been described in detail (Hafke et al., 2005). Microelectrodes were pulled from aluminosilicate microcapillaries with an outer diameter of 1 mm and an internal filament (SM100F-10, Harvard Apparatus LTD, Edenbridge, Kent, UK) on a vertical electrode puller (GETRA, München, Germany). The tip diameter of these electrodes was 0.5–1 μm. The microelectrodes were back-filled with 500 mM KCl and clamped in an Ag/AgCl pellet electrode holder (WPI, Sarasota FL, USA). The microelectrode was connected to the probe of the amplifier (DUO 773 high-input impedance differential electrometer, WPI, Sarasota FL, USA). The Ag/AgCl reference electrode was connected to the bathing medium by a 2% agar bridge (w/v) filled with 500 mM KCl solution (Hafke et al., 2005). After incubation in BM for 1 h, microelectrodes were impaled into phloem cells under microscopic surveillance (Hafke et al., 2005). All measurements were performed at a room temperature of 23–25°C. Membrane potentials of sieve elements (SEs) and adjacent phloem parenchyma cells (PPCs) were recorded either in main veins or in internodes along the plant. The term plant length index (PLI) was introduced to define the recording positions. The main vein of youngest mature leaf was defined as PLI 1, a position half-way along the stem as PLI 0.5, and the position at the transition between the autotrophic and heterotrophic part of the stem as PLI 0 (Figure 1A).

#### Measurement of sucrose-induced depolarizations in intact phloem tissue

Various sucrose concentrations (1–100 mM), dissolved in BM, were administered to the bare-lying phloem tissue. Because the membrane potential measurements – in particular those of PPCs - are extremely sensitive to perfusion turbulence, sucrose solutions were supplied via a microcapillary (20–30 μm in diameter) mounted on a micromanipulator and connected to a pressure-driven microinjector (Cell Tram Oil microinjector, Eppendorf, Hamburg, Germany).

Sucrose-induced depolarizations (e.g., Lichtner and Spanswick, 1981) were plotted against the sucrose concentration. *K*_*m*_ -values for sucrose uptake by SE/CCs or PPCs for the uptake models MM (single Michaelis–Menten term) and 2 MM (two Michelis-Menten terms) were obtained by non-linear least square fitting (NLSF) using SIGMA PLOT 11.2 software package (Systat Software, San Jose, CA). Goodness-of-fit was judged from *R*^2^ and the sum of squared residuals (SSR). A smaller SSR is indicative of a tight fit of the model to the data (e.g., Ritchie and Prvan, 1996; Motulsky and Christopoulos, 2003). The formulas applied are exemplified by the following biphasic MM equation for two active transport systems (2 MM terms) (1):

(1) $$\Delta V=\frac{\Delta V_{1}{\left[\text{suc}\right]}_{\text{ext}}}{K_{m1}+{\left[\text{suc}\right]}_{\text{ext}}}+\frac{\Delta V_{2}{\left[\text{suc}\right]}_{\text{ext}}}{K_{m2}+{\left[\text{suc}\right]}_{\text{ext}}}$$

in which ΔV = amplitude of depolarization, ΔV_1_, ΔV_2_ the maximum depolarization in analogy to *V*_max_ and *K*_*m*_ describes the MM constant of the transport system. Here, *K*_*m*_ is the concentration at which 50% of the maximal depolarization is reached. *K*_*m*_ values were also calculated from Eadie–Hofstee (EH) transformations (Hofstee, 1959) assuming either a single MM (one slope; -*K*_*m*_) or a biphasic 2 MM kinetic (two different slopes, -*K*_*m*1_, -*K*_*m*2_). The EH transformation for one slope is given by (2):

(2) $$\Delta V=\Delta V_{\max}-K_{m}\frac{\Delta V}{{\left[\text{suc}\right]}_{\text{ext}}}$$

#### Model selection using akaike's information criterion (AIC)

Akaike's (entropy-based) Information Criterion (Akaike, 1973, cited in Burnham and Anderson, 2004) is an approach to select the best model from a set of models. The AIC determines how well the data supports each model (for details please see Motulsky and Christopoulos, 2003; Burnham and Anderson, 2004). It has an in-built penalty for models which larger number of parameters (more complex models). The model which fits well but has a minimum number of parameters (simplicity and parsimony) is preferred (Burnham and Anderson, 2004). In the present work AIC-based model selection was used to discriminate between single MM or double MM (2 MM) sucrose-uptake kinetics.

For a quantitative comparison of different models, an AIC value is determined for each model. For small sample sizes, Akaike's second-order information criterion (AIC_c_; e.g., Motulsky and Christopoulos, 2003; Burnham and Anderson, 2004) is used for calculation which is given by Equation (3):

(3) $${\text{AIC}}_{\text{c}}=N\text{In}\frac{\text{SSR}}{N}+2K+\frac{2K(K+1)}{N-K-1}$$

where N is the number of data points, SSR is the sum of squared residuals obtained from non-linear least-square fittings (either to the model MM or 2 MM) to the data points and K is the number of parameters to be fitted plus one (Motulsky and Christopoulos, 2003). The number of parameters is 2 for MM and 4 in case of 2 MM. The model with the lower AIC value is more likely to be correct. If AICc values are very close or equal, there is no evidence to prefer one model over the other or each model is equally likely to be correct (Motulsky and Christopoulos, 2003). For a more quantitative comparison of models, the strength of evidence for each model is determined by calculating Δ_*i*_ (= delta AIC) and Akaike's weight*w*_*i*_, respectively. Δ_*i*_ is simply calculated by subtraction of the AIC_c_ value for the best model (= minimum AIC_c_ value) from the AIC_c_ value of the other model(s) (e.g., Burnham and Anderson, 2004). Δ_*i*_ are then transformed into Akaike's weight (4):

(4) $$w_{i}=\frac{e^{-0.5\Delta i}}{1+e^{-0.5\Delta i}}$$

Akaike weights (*w*_*i*_) are the “weight of evidence” in favor of a certain model being the best model in the candidate set (e.g., Burnham and Anderson, 2004). From Akaike's weight, an evidence ratio of probabilities can be calculated by (5):

(5) $$\text{Evidence ratio}=\frac{\text{Probabilty that model 1 is correct}}{\text{Probabilty that model 2 is correct}}=\frac{1}{1+e^{-0.5\Delta_{i}}}$$

The evidence ratio indicates how many times more likely is one model compared to the other (Motulsky and Christopoulos, 2003).

#### Isolation of sieve-element protoplasts and patch-clamp recordings

Sieve-element protoplasts were isolated according to Hafke et al. (2007). Internodes from 3 to 4 weeks old plant were excised and split longitudinally. For coarse mechanical isolation of phloem strands, vascular tissues were sliced (slice thickness ~300 μm) with a razor blade from the cut tangential face of the internode. The slices were transferred into an enzymatic wall digestion medium (Hafke et al., 2007) and incubated over night (approx. 10 h) at room temperature. Disintegrating phloem strands were filtered through an 80 μm nylon mesh, washed two times and stored in a medium containing 500 mM mannitol, 2 mM KCl, 1 mM CaCl_2_, 1 mM MgCl_2_, 2.5 mM MES/NaOH, pH 5.7. Only simple sieve-element protoplasts that were identified by inclusion of forisomes, were used for patch clamp experiments (cf. Hafke et al., 2007)

A cytosolic solution (pipette solution in contact with the cytoplasmic side of the protoplast) was slightly modified in comparison to Carpaneto et al. (2005) and contained 30 mM KCl, 1 mM EGTA, 3 mM MgCl_2_, 5 mM sucrose, 400 mM mannitol, 10 mM BTP (bis-tris-propane), titrated with MES to pH 7.5. Protoplasts were bathed in a solution composed of 2 mM MgCl_2_, 1 mM CaCl_2_, 1 mM GdCl_3_, 450 mM mannitol, 10 mM MES, titrated with BTP to pH 5.5. For sucrose application, 450 mM mannitol was replaced by 350 mM mannitol and 100 mM sucrose as compared to the bathing solution. This solution was delivered in the vicinity of the protoplast via a microcapillary connected to a pressure-driven microinjector.

Patch-clamp recordings on sieve-element protoplasts were performed in the whole-cell configuration as described before (cf. Hafke et al., 2007). Whole-cell currents were recorded with an A-M Systems patch-clamp amplifier (Model 2400, A-M Systems, Inc.®, Carlsborg, USA), filtered with an eight-pole Bessel filter at either 0.5 or 1 kHz and stored online on a personal computer at a 2,5/5 kHz sampling frequency via a Digidata 1440A interface digitizer (Molecular Devices, MDS Analytical Technologies, Sunnydale, California, USA). Data were low-pass filtered off-line at 10 or 25 Hz. Data recording, acquisition and analysis were performed with pCLAMP 10 hardware and software facilities (Molecular Devices Corporation, Sunnydale, California, USA). For comparison of individual protoplasts, current amplitudes were normalized to the whole-cell membrane capacitance. Membrane capacitance was determined from capacitive currents measured in response to short (10 ms) voltage steps of 10 mV (Gillis, 1995). All command voltages were corrected off-line for liquid junction potential (Neher, 1992) using a Liquid Junction Potential Calculator (Ng and Barry, 1995). All measurements were carried out at room temperatures of about 21°C.

## Results

### Membrane potentials of sieve element/companion cell complex (SE/CCS) and phloem parenchyma cells (PPCS) along the plant axis

Under the usually prevailing source-limiting conditions, the dynamic sucrose exchange along the transport phloem is controlled by pmf-driven transporters located at the plasma-membranes of SE/CCs and PPCs. Earlier studies pointed out that competitiveness between SEs and PPCs is determined by the electric component of the pmf (Hafke et al., 2005). Hence, membrane potentials of SE/CCs and PPCs were determined along the axis of intact *Vicia faba* plants after impalement by microelectrodes under microscopic surveillance as illustrated previously (Hafke et al., 2005).

To standardize the electrode positions along plants of various lengths, the plant length index (PLI) was introduced (Figure 1A). The position on the main vein of the youngest mature leaf was set to be PLI 1, a position halfway along the stem was defined as PLI 0.5 and the transition from the heterotrophic to the autotrophic region just above ground level corresponds to PLI 0. The resting potentials of SE/CCs and PPCs were recorded at the respective plant axis positions and the membrane potential ratios *V*_m SE/CC_/*V*_m PPC_ calculated (Figure 1B).

The average membrane potentials of SE/CCs (Figure 1B) varied between −127 mV at PLI 1 and −112 mV at PLI 0 and those of the PPCs remain around −100 mV along the entire stem stretch. The distinct and consistent difference between the membrane potentials of SE/CCs and PPCs at various PPIs indicated a symplasmic disjunction as found before in transport phloem (Oparka et al., 1994; van Bel and van Rijen, 1994; Kempers et al., 1998; Hafke et al., 2005). Since the membrane potentials of the SE/CCs declined more rapidly along the plant axis than those of the PPCs (see ratio, Figure 1B), the relative competitiveness of the PPCs may increase towards the stem base.

Membrane potentials were also recorded in SEs and PPCs embedded in free-lying vascular root tissue. There, the SE membrane potentials are drastically reduced to −55 ± 2.4 mV (*n* = 4) in comparison with SEs in the stem. No stable values for PPCs could be obtained so that no conclusions could be drawn regarding the relative competitiveness between SEs and PPCs in roots.

### *K*_*m*_ values of sucrose uptake in either SE/CCS or PPCS measured by sucrose-induced depolarization of membrane potentials

As discussed before (Hafke et al., 2005), kinetics of sucrose uptake by SE/CCs and PPCs is a key element in the competitiveness between SE/CCs and PPCs and, hence, for photoassimilate partitioning. However, the cell-specific uptake kinetics has not been determined conclusively to date. Studies of ^14^C-sucrose uptake by phloem strips do not discriminate between the kinetics of SE/CCs and PPCs. Furthermore, uptake studies using SE- and PPC-protoplasts would require an immense amount of protoplasts which is virtually impossible given the painstaking isolation and identification procedures. Therefore, we measured membrane depolarizations of single cells induced by various apoplasmic sucrose concentrations under physiological conditions (e.g., Lichtner and Spanswick, 1981). Natural apoplasmic sucrose concentrations are in the range between 1.0 and 60.0 mM (cf. Patrick and Turvey, 1981; Minchin and Thorpe, 1984; Voitsekhovskaja et al., 2000; Kang et al., 2007).

As reported before (Hafke et al., 2005), microelectrodes were impaled into SEs (Figures 1C,D) or PPCs (Figure 1G). Following stabilization of the membrane potential, various sucrose concentrations were supplied to SE/CCs by bath perfusion (for SEs) or local micropipette-mediated solute administration (for PPCs). Electrical recordings showed graded effects of various sucrose concentrations on the membrane potentials of SEs (Figure 1C) and PPCs (Figure 1G). As a general pattern, a fast sucrose-induced depolarization brought about by H^+^ co-transport (e.g., Carpaneto et al., 2005) was followed by a gradual repolarization. Replacement of the sucrose solutions by an iso-osmotic mannitol solution accelerated the repolarization to the resting potential (Figure 1C).

At a decreased external pH (3.5 instead of 5.7), application of the same sucrose concentration (10 mM) resulted in a 3-fold increase in the magnitude of depolarization (Figure 1D) which is in agreement with the role of protons in pmf-driven H^+^-sucrose symport (e.g., Delrot and Bonnemain, 1981; Reinhold and Kaplan, 1984).

The relationship between sucrose concentration and SE or PPC depolarization can be described by single Michaelis–Menten kinetics (MM) or a biphasic Michaelis–Menten kinetics (2 MM) (Figures 1E,H). Regression diagnostics (sum of squared residuals SSRs, *R*^2^) from non-linear least-square data fitting (NLSF) revealed a slightly better fit to 2 MM as indicated by smaller SSRs and a higher *R*^2^ as compared to MM (Table 1). Furthermore Eadie–Hofstee (EH) transformations revealed two linear components (Figures 1F,I) which are indicative for two separate, simultaneously operating uptake systems (e.g., Reinhold and Kaplan, 1984). The *K*_*m*_ values of either uptake system can be calculated from the linear regression at low and high sucrose concentrations.Unmistakable biphasic uptake kinetics are visible for the SE data at PLI 0.5 (Figure 1F, Table 1), but those at PLI 1 and PLI 0, data can be interpreted to be due to either MM or 2 MM (Figure 1F, Table 1).

**Table 1:**
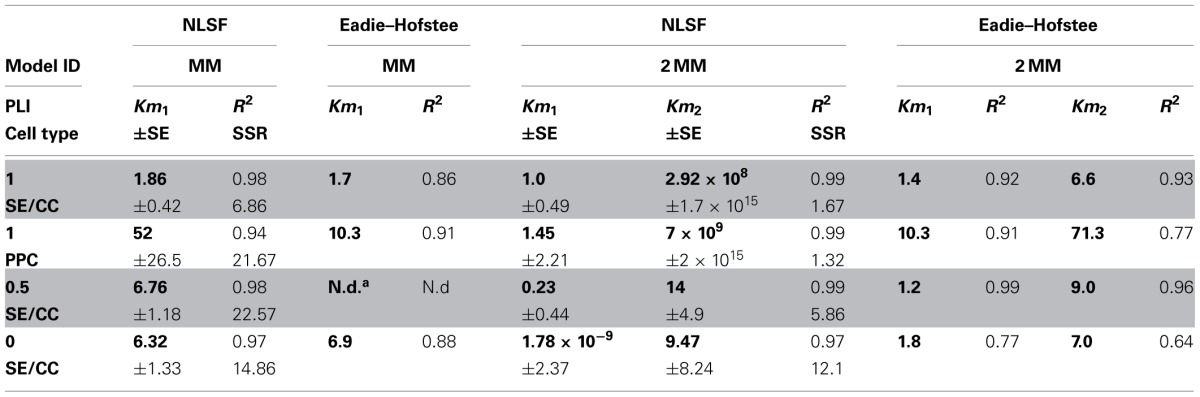
**Summary of *K*_*m*_-values for sucrose uptake into SE/CCs or PPC obtained by non-linear least-square fitting (NLSF) using SIGMA PLOT 11.2 or Eadie–Hofstee transformation**.

*Sucrose-uptake parameters of the concentration-dependence of the sucrose-induced depolarizations were fitted to a Michaelis–Menten equation (MM) and the sum of two MMs (2 MM). K_m_ values were also obtained from the linear fittings of Eadie–Hofstee transformations (MM, 2 MM). Goodness-of-fit was evaluated from R^2^ and SSR. A small SSR is indicative of a tight fit of the model to the data. Standard errors (SE) of the fitted parameters are given for the non-linear fittings*.

*^a^N.d.: not determined. Given the distinct biphasic character of the EH transformation, a linear regression of the data points with only one K_m_ value is not appropriate*.

Diagnostic tools like SSR and *R*^2^ values alone do not allow predictions on the adequacy of a kinetic model (MM or 2 MM). Akaikes Information Criterion (AIC, Motulsky and Christopoulos, 2003; Burnham and Anderson, 2004) was applied to test for the best model (Table 2). MM is more likely to be correct then 2 MM which is indicated by smaller AIC_c_ values for the MM model (Table 2). The small difference in Δ_*I*_ of <2 at PLI 0.5 (1.82) indicates that 2 MM has a substantial support (evidence) whereas models having Δ_*I*_ > 10 (in case of 2 MM at PLI1 and PLI0) have essentially no support (Burnham and Anderson, 2004). Fitting the data to MM would render a shift in *K*_*m*_ values for SEs along the plant axis (Table 1). *K*_*m*_ values obtained by NLSF increased from 1.86 mM (EH: 1.73 mM) at PLI1 to 6.32 mM (EH: 9 mM) at PLI 0 (Table 1) suggesting a decreased sucrose retrieval by SEs along the plant axis.

**Table 2:**
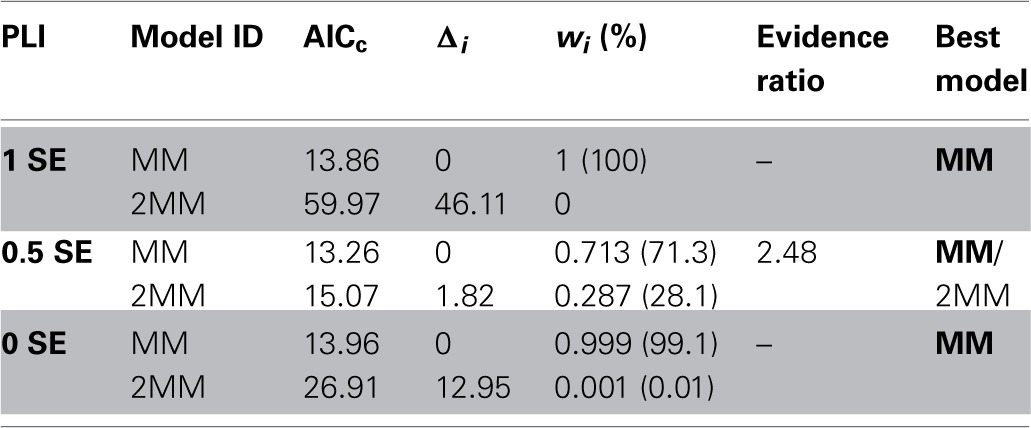
**Comparing and selecting different models (MM, 2 MM) by Akaike's second-order information criterion**.

*Denotations: AICc: AIC value, Δ_i_: delta AIC_c_ W_i_: Akaike's weight. For the definitions of the AIC parameters please see Methods section*.

For PPCs, a *K*_*m*_ value of 52 ± 26.5 mM showing a high standard error was calculated (Table 1) when MM was adopted as the mode of uptake. Using 2 MM, Eadie-Hofstee calculations produced *K*_*m*1_ and *K*_*m*2_ values of 10 and 70 mM, respectively, while NLSF revealed a *K*_*m*1_ value of 1.45 mM and infinitesimal high *K*_*m*2_ (Table 1). Irrespective of the mode(s) of uptake, it suggests that the affinity for sucrose uptake is much lower in PPCs than in SEs.

### Measurability of sucrose/H^+^ transporter activity in the plasma membrane of sieve-element protoplasts

**Figure 2:**
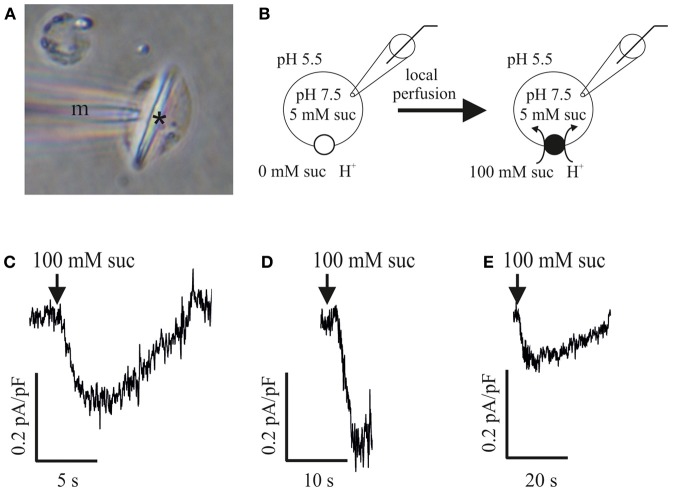
**Patch clamp recordings of sucrose-induced H^+^ currents in sieve-element (SE) protoplasts of *Vicia faba***. **(A)** SE protoplast containing a forisome (asterisk) with microcapillary (m) attached to the plasma membrane immediately before a patch-clamp experiment. **(B)** Experimental patch-clamp conditions with 5 mM sucrose at the inner side of an SE protoplast (pipette pH 7.5) and 0 mM sucrose at the beginning of the experiment (left) and 100 mM sucrose during perfusion (right) at the outer side (bath pH 5.5). **(C–E)** Three independent measurements of sucrose-induced H^+^-currents in SE protoplasts. Membrane voltage was clamped to −106 mV. Current traces showing a downward deflection (increase in inwardly directed currents) in response to 100 mM sucrose application (arrows). All currents were normalized to the membrane capacitance C_m_.

In analogy to studies of the phloem-localized sucrose carrier ZmSUT1 expressed in *Xenopus* oocytes (Carpaneto et al., 2005), patch-clamp technique was applied to SE protoplasts in the whole-cell configuration (cf. Hafke et al., 2007) for a direct detection of the sucrose/H^+^ transporter activity. SE protoplasts (Figure 2A) were bathed in a sucrose-free medium (pH 5.5; Figure 2B) and clamped to a holding voltage of −106 mV. In response to external supply of 100 mM sucrose (pH 5.5) by local bath perfusion, an increase in inward current (positive current into the cell, cf. Bertl et al., 1992) was recorded. Normalized currents were in the range of −0.1 to −0.3 pA/pF (−0.2 ± 0.07 pA/pF, *n* = 4). This time-dependent increase in negative currents (downward deflections, Figures 2C–E) represent proton currents generated by a H^+^/sucrose symporter mediating sucrose transport into SEs as reported for ZmSUT1 expressed in *Xenopus* oocytes (Carpaneto et al., 2005).

## Discussion

### *In situ* determination of *K*_*m*_ values for sucrose uptake by phloem cells using electrophysiology

Kinetic parameters for sucrose uptake are crucial for the competition between SE/CCs and PPCs in transport phloem and the distribution of photoassimilates over axial and terminal sinks (van Bel, 1996; Hafke et al., 2005). Other than in previous approaches, *K*_*m*_ values for sucrose uptake were calculated here by measuring sucrose-induced membrane depolarizations of SEs and PPCs (Figure 1) in analogy to an approach using cut aphid stylets in combination with electrophysiology (Wright and Fisher, 1981). As aphid stylets are solely inserted into SEs, the latter technique is not applicable for PPC recordings.

**Figure 3:**
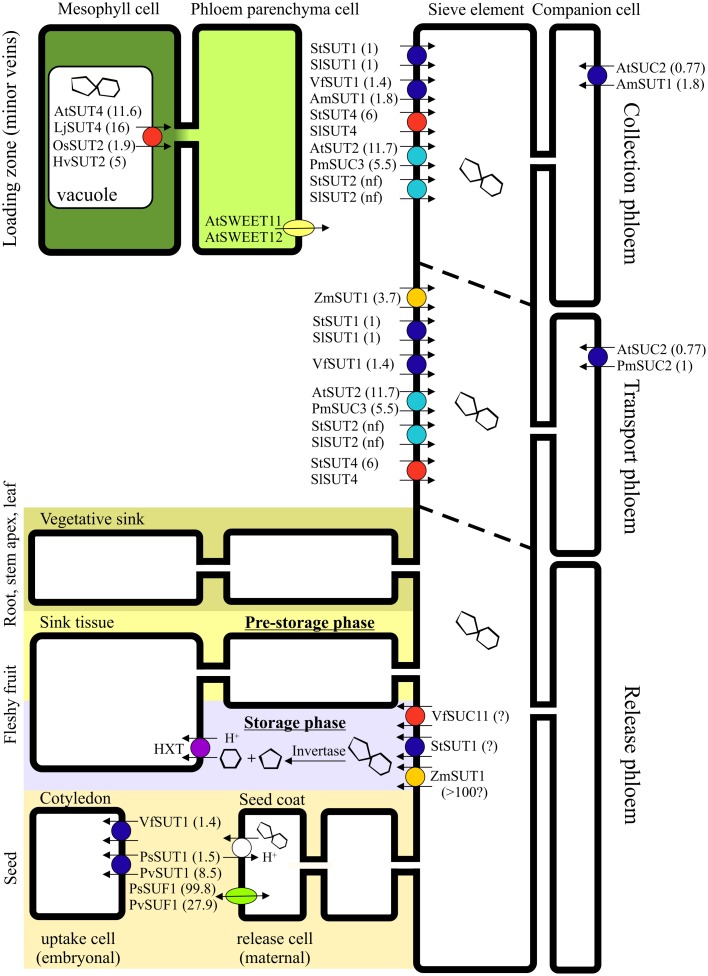
**Schematic presentation of the affinity constants of sucrose transporters involved in apoplasmic phloem loading, transport, and unloading**. *K*_*m*_ values were taken from (Riesmeier et al., 1993; Weber et al., 1997; Barker et al., 2000; Schulze et al., 2000; Weise et al., 2000; Weschke et al., 2000; Manning et al., 2001; Reinders et al., 2002; Barth et al., 2003; Knop et al., 2004; Carpaneto et al., 2005; Zhou et al., 2007; Eom et al., 2011; Gould et al., 2012). Sucrose transporters are indispensible for apoplasmic phloem loading, but play a rather marginal part in symplasmic phloem loading as it occurs in numerous species. In transport phloem, sucrose transporters are prominent under the usually prevailing source-limiting conditions, whereas their role is reduced under sink-limiting conditions. While phloem unloading in terminal leaf and root sinks occurs entirely symplasmically, the unloading path in larger sinks may include an apoplasmic step. Due to the obligatory symplasmic isolation of the embryo from the maternal seed-coat tissue in *Pisum sativum* and *Phaseolus vulgaris*, an apoplasmic loading step is required involving sucrose transporters and facilitators (Patrick, 2013). A sucrose/proton antiport mechanism of unknown identity was postulated in seed coats of *Vicia faba* (Fieuw and Patrick, 1993) and *Phaseolus vulgaris* (Walker et al., 1995). PsSUT1 was also localized in the vascular tissue of the seed coat, where it is assumed to play a role in sucrose efflux (Zhou et al., 2007). In fleshy fruits, phloem unloading often follows diverse routes in dependence of the developmental stage. After symplasmic unloading during the pre-storage phase, phloem unloading is assumed to occur apoplasmically during the storage phase (Ruan and Patrick, 1995; Zhang et al., 2006; Nie et al., 2010; Tegeder et al., 2013), while apoplasmic sieve-element unloading precedes symplasmic transport in the post-sieve-element pathway during the entire development of apples (Zhang et al., 2004). In walnuts, the pathway of unloading depends on the target tissue: photoassimilates are transported symplasmically to the seed coat, but apoplasmically to the fleshy pericarp (Wu et al., 2004). In potato tubers, apoplasmic unloading switches to symplasmic unloading during development (Viola et al., 2001). During the first phase of stolon development, the sucrose transporter StSUT1 is assumed to be involved in sucrose unloading form the phloem acting as a sucrose efflux transporter (Kühn et al., 2003). Sucrose transporters of the SUT1 clade are colored in blue, of the SUT2 clade in cyan, of the SUT3 clade in orange, whereas transporters of the SUT4 clade are colored in red (according to the phylogenetic classification by Kühn and Grof, 2010). *K*_*m*_ values (mM) of the transporters are displayed in brackets.

The sucrose-induced depolarizations are consistent with H^+^-sucrose co-transport with a 1:1 stoichiometry (e.g., Lichtner and Spanswick, 1981; Wright and Fisher, 1981; Carpaneto et al., 2005). The magnitude of depolarization varies with external sucrose concentrations in keeping with Michaelis–Menten uptake kinetics (Figures 1E,H). The *K*_*m*_ values calculated for uptake by sieve elements (Table 1) match well with those obtained by uptake studies in oocytes and yeast (Figure 3) which can be regarded as proof for the adequacy of the present electrophysiological approach. The *K*_*m*_ values (Table 1) are higher than those observed for sucrose uptake by isolated *Commelina* minor veins (0.5 mM), but determination of the parameters of active uptake may have been confounded by an appreciable diffusional uptake component in this study (van Bel and Koops, 1985).

### Selection of the kinetic model

Plotting the relationship between sucrose concentration and membrane depolarization can be described by either single (MM) or biphasic Michaelis–Menten kinetics (2 MM). As for SEs, Eadie-Hofstee transformations revealed some evidence in favor of two uptake systems (Figure 1F, Table 1), but the differences in significance between MM or 2 MM were marginal. To distinguish between MM and 2 MM, Akaike's Information Criterion (AIC)-based model selection was invoked for further analysis (Table 2). As a result, MM is favored for SEs at PLI 1 and PLI 0 (Table 2), whereas AIC supported also 2 MM for SEs at PLI 0.5 (see also Figure 1F) by a difference in AIC_c_ values between MM and 2 MM of Δ_*i*_ < 2 (e.g., Burnham and Anderson, 2004).

The inconclusive assessment of the uptake mode(s) which may also be due to the limited data sets, leaves us with two possible interpretations:

- Only one single active uptake system is involved, but MM kinetics is distorted by artifacts induced by the experimental approach. Uptake may be influenced by unstirred layers in vicinity of the plasma membrane as described for intestinal studies (Thomson, 1977; Thomson and Dietschy, 1977; Gardner and Atkinson, 1982). The absorption of solutes in anatomical complex tissues leads to concentrations in the close vicinity of the cell membrane being lower than that in the bulk fluid leading to a deformation of the Michaelis–Menten kinetic or the Eadie-Hofstee transformation and a serious shift in *K*_*m*_ of the sucrose uptake system to higher values (Winne, 1977; Thomson, 1977; Thomson and Dietschy, 1977; Gardner and Atkinson, 1982). Diffusion through the cell-wall microfibrils may be limited by physical forces in the cell-wall micro-environment.
  A further pitfall causing dual kinetics may be symplasmic coupling in intact tissues: biphasic kinetics could be ascribed to differential contributions of electrically coupled cells. Therefore, symplasmic isolation is an absolute prerequisite for a correct interpretation of the data. Fluorochrome studies demonstrated that SE/CCs in transport phloem are strictly disjunct from PPCs under the present conditions (van Bel and van Rijen, 1994; Patrick and Offler, 1996; Knoblauch and van Bel, 1998; Hafke et al., 2005), when the few plasmodesmata in the SE/CC-PPC interface (Kempers et al., 1998) are closed. By contrast, SEs and CCs are strongly coupled *in situ* via numerous PPUs (Kempers et al., 1998). However, electrode impalement likely confers occlusion by PPUs (Knoblauch and van Bel, 1998) so that the biphasic depolarization patterns are to be merely ascribed to SEs. For PPCs, it remains uncertain to which degree plasmodesmata towards adjacent cells are closed after microelectrode insertion.
- Two active systems are involved in sucrose uptake. Several studies postulated a second linear component in addition to active uptake resulting in biphasic kinetics (e.g., van Bel and Koops, 1985; Daie, 1987). This linear uptake was assigned to facilitated diffusion, but could be equally well the linear section of a low-affinity MM system. The latter opinion was corroborated by PCMS inhibition and FC stimulation of this linear component of sucrose uptake (A. J. E. van Bel and M. Tjaden, unpublished results). Active uptake by a low-affinity system would be in line with the continuously increasing depolarizations at higher sucrose concentrations (Figures 1E,H).

### Consequences for carbohydrate processing in intact plants

The uptake mode(s) has(have) a strong impact on sucrose processing along the phloem pathway. The existence of one sucrose uptake system in SEs of transport phloem would disclose a shift in the *K*_*m*_ values of SEs from 1.8 mM to approx. 6 to 7 mM downwards along the plant axis (Table 1, NLSF, EH for MM). In parallel with the increase in the affinity constant, membrane potentials in SEs decline along the plant axis (Figure 1A), leading to reduced pmf-driven sucrose uptake (e.g., Hafke et al., 2005). A comparable voltage-dependence of *K*_*m*_ values was already characterized for the sucrose carrier ZmSUT1 expressed in oocytes (Carpaneto et al., 2005). Carpaneto et al. (2005) showed, that that *K*_*m*_ values for ZmSUT1 increased at more positive membrane voltages. For PPCs showing much higher *K*_*m*_values for sucrose uptake (>10 mM; Table 1), more detailed data analysis on kinetic properties of sucrose uptake is necessary. Recently, two efflux carriers mediating a key step in phloem loading have been characterized in Arabidopsis: AtSWEET11 and AtSWEET12. These two sucrose transporters are highly expressed in leaves and are both localized in the plasma membrane of phloem parenchyma cells (Chen et al., 2012). The *atsweet11*/*atsweet12* double insertional mutant plants are defective in phloem loading and display a phenotype similar to AtSUC2 knock-out mutants with regard to sugar and starch accumulation (Gottwald et al., 2000). Both AtSWEET11 and AtSWEET12 seem to be different from the other proteins belonging to the SWEET family because of their low affinity to sucrose, with the *K*_*m*_ values for an influx ≈70 and efflux >10 mM (AtSWEET12). Because of the pH-independent transport, it was suggested that the sucrose translocation may rely on a uniport system (Chen et al., 2012).

It should be noted, that, pmf-driven uptake rates may be constant, since membrane potentials of PPCs did not change along the plant axis (Figure 1B). Both membrane potential gradients and *K*_*m*_ gradients infer that the competitiveness of sieve elements declines downwards along the axis.

Carbohydrate partitioning and management strongly depend on the release/retrieval of photoassimilates along the phloem path under source-limiting conditions (Patrick and Offler, 1996; van Bel, 2003a,b; Hafke et al., 2005). The rate of (temporary) diversion of photoassimilates to axial sinks is determined by a competition between SE/CCs and PPCs (Hafke et al., 2005). According to the present data, the relative competitiveness of PPCs tends to increase near the stem basis (ratio, Figure 1B). According to the *K*_*m*_-values assuming one sucrose uptake system along the axis (Tables 1, 2), the competitiveness of SE/CCs is higher at low apoplasmic sucrose concentrations than at high concentrations which can be handled more easily by PPCs (having low-affinity uptake system). Under sink-limiting conditions, the amounts of photoassimilates available for the axial sinks are extremely high. Then, membrane uptake systems will be of minor importance since most of the photoassimilates move through open plasmodesmata towards the PPCs (Patrick and Offler, 1996). Under these circumstances, the low-affinity systems of PPCs may be quite useful for retrieval of massive amounts of sucrose leaking from the axial sink cells.

### Patch-clamp recordings of sucrose-induced proton fluxes in sieve-element protoplasts

In previous studies, ZmSUT1 was heterologously expressed in oocytes and characterized functionally in detail using a patch-clamp approach (Carpaneto et al., 2005, 2010). The sucrose-coupled proton currents were reversible depending on the direction of the sucrose and pH gradients and the apparent affinity constant *K*_*m*_ of ZmSUT1 exhibited a pronounced voltage and pH-dependence (Carpaneto et al., 2005). The turnover rate of ZmSUT1 at a physiological membrane voltage of −120 mV is about 500 s^−1^ (Carpaneto et al., 2010). Based on this value, a transporter density of 10^4^/μm^2^ was calculated for oocytes expressing ZmSUT1 (Carpaneto et al., 2010), which exceeds by far the transporter densities to be expected in plasma membranes under natural conditions.

Using a similar patch-clamp approach (cf. Carpaneto et al., 2005), a sucrose/H^+^ symport activity was detected in the plasma membrane of SE protoplasts (Hafke et al., 2007) as indicated by an increase in inwardly directed currents (visible as downward deflections relative to the baseline) in response to sucrose supply. Neglecting possible rundown effects previously described for the H^+^/sucrose symporter ZmSUT1 (Carpaneto et al., 2005), negative currents with current densities I/C_m_ around −0.2 pA/pF (Figure 2) were recorded in the presence of 100 mM sucrose, a membrane voltage of −106 mV and a gradient of 2 pH units. The observed current densities lie in the order of magnitude of sucrose-induced H^+^ currents (Schulz et al., 2011; SUC4 transporters) or myo-inositol driven H^+^ currents (Schneider et al., 2008) in vacuoles of *Arabidopsis thaliana*. To date, sucrose carrier activity in native membrane systems were measured solely in isolated vacuoles by the patch-clamp technique (Schulz et al., 2011). Given the detectable H^+^/sucrose symporter activity in the plasma membrane of SEs (Figure 2), SE protoplasts might become a suitable tool to characterize sucrose transporters in their natural membrane environment and hence complement the data set obtained with transporters expressed in heterologous systems like oocytes (Carpaneto et al., 2005, 2010).

### Conflict of interest statement

The authors declare that the research was conducted in the absence of any commercial or financial relationships that could be construed as a potential conflict of interest.

## References

[B1a] Akaike H. (1973). Information theory as an extension of the maximum likelihood principle, in Second International Symposium on Information Theory, eds Petrov B. N. Csaki F. (Budapest: Akademiai Kiado), 267–281

[B1] Ayre B. (2011). Membrane-transport systems for sucrose in relation to whole-plant carbon partitioning. Mol. Plant 4, 377–394 21502663 10.1093/mp/ssr014

[B2] Barker L. Kühn C. Weise A. Schulz A. Gebhardt C. Hirner B. (2000). SUT2, a putative sucrose sensor in sieve elements. Plant Cell 12, 1153–1164 10899981 10.1105/tpc.12.7.1153 PMC149056

[B3] Barth I. Meyer S. Sauer N. (2003). PmSUC3: characterization of a SUT2/SUC3-type sucrose transporter from *Plantago major*. Plant Cell 15, 1375–1385 12782730 10.1105/tpc.010967 PMC156373

[B4] Bertl A. Blumwald E. Coronado R. Eisenberg R. Findlay G. Gradmann D. (1992). Electrical measurements on endomembranes. Science 258, 873–874 1439795 10.1126/science.1439795

[B5] Burnham K. P. Anderson D. R. (2004). Multimodel inference: understanding AIC and BIC in model selection. Soc. Methods Res. 33, 261–304

[B6] Carpaneto A. Geiger D. Bamberg E. Sauer N. Fromm J. Hedrich R. (2005). Phloem-localized, proton-coupled sucrose carrier ZmSUT1 mediates sucrose efflux under the control of the sucrose gradient and the proton motive force. J. Biol. Chem. 280, 21437–21443 15805107 10.1074/jbc.M501785200

[B7] Carpaneto A. Koepsell H. Bamberg E. Hedrich R. Geiger D. (2010). Sucrose- and H^+^-dependent charge movements associated with the gating of sucrose transporter ZmSUT1. PLoS ONE 5:e12605 10.1371/journal.pone.0012605 20838661 PMC2935479

[B8] Chen L. Q. Qu X. Q. Hou B. H. Sosso D. Osorio S. Fernie A. R. (2012). Sucrose efflux mediated by SWEET proteins as a key step for phloem transport. Science 335, 207–211 22157085 10.1126/science.1213351

[B9] Daie J. (1987). Sucrose uptake in isolated phloem of celery is a single saturable transport system. Planta 171, 474–482 24225708 10.1007/BF00392294

[B10] Delrot S. Bonnemain J.-L. (1981). Involvement of protons as a substrate for the sucrose carrier during phloem loading in *Vicia faba* leaves. Plant Physiol. 67, 560–564 16661714 10.1104/pp.67.3.560 PMC425725

[B11] Eom J. S. Cho J. I. Reinders A. Lee S. W. Yoo Y. Tuan P. Q. (2011). Impaired function of the tonoplast-localized sucrose transporter in rice, OsSUT2, limits the transport of vacuolar reserve sucrose and affects plant growth. Plant Physiol. 157, 109–119 21771914 10.1104/pp.111.176982 PMC3165862

[B12] Eschrich W. Evert R. F. Young J. H. (1972). Solution flow in tubular semipermable membranes. Planta 107, 279–300 10.1007/BF00386391 24477479

[B13] Fieuw S. Patrick J. W. (1993). Mechanisms of photosynthate efflux from *Vicia faba* L. seed coats. J. Exp. Bot. 44, 65–74

[B14] Gardner M. L. G. Atkinson G. L. (1982). Kinetic analysis of transport processes in the intestine and other tissues. Clin. Sci. 63,405–414 6749382 10.1042/cs0630405

[B15] Geiger D. (2011). Plant sucrose transporters from a biophysical view. Mol. Plant 4, 395–406 21502662 10.1093/mp/ssr029

[B16] Gillis K. D. (1995). Techniques for membrane capacitance measurements, in Single Channel Recordings, eds Sakmann B. Neher E. (New York, NY: Plenum Press), 155–197

[B17] Gottwald J. R. Krysan P. J. Young J. C. Evert R. F. Sussman M. R. (2000). Genetic evidence for the *in planta* role of phloem-specific plasma membrane sucrose transporters. PNAS 97, 13979–13984 11087840 10.1073/pnas.250473797 PMC17686

[B18] Gould N. Thorpe M. R. Minchin P. E. H. (2004). Direct measurements of sieve element hydrostatic pressure reveal strong regulation of sieve element hydrostatic pressure after pathway blockage. Funct. Plant Biol. 31, 987–993 10.1071/FP04058 32688967

[B19] Gould N. Thorpe M. R. Pritchard J. Christeller J. T. Williams L. E. Roeb G. (2012). AtSUC2 has a role for sucrose retrieval along the phloem pathway: evidence from carbon-11 tracer studies. Plant Sci. 188–189, 97–101 22525249 10.1016/j.plantsci.2011.12.018

[B20] Hafke J. B. van Amerongen J. K. Kelling F. Furch A. C. U. Gaupels F. van Bel A. J. E. (2005). Thermodynamic battle for photosynthate acquisition between sieve tubes and adjoining parenchyma in transport phloem. Plant Physiol. 138, 1527–1537 15980202 10.1104/pp.104.058511 PMC1176423

[B21] Hafke J. B. Furch A. C. U. Reitz M. U. van Bel A. J. E. (2007). Functional sieve element protoplasts. Plant Physiol. 145, 703–711 17885083 10.1104/pp.107.105940 PMC2048790

[B22] Hofstee B. H. J. (1959). Non-inverted versus inverted plots in enzyme kinetics. Nature 184, 1296–1298 14402470 10.1038/1841296b0

[B23] Kang Y. Outlaw W. H. Jr. Fiore G. B. Riddle K. A. (2007). Guard cell apoplastic photosynthate accumulation corresponds to a phloem-loading mechanism. J. Exp. Bot. 58, 4061–4070 18182421 10.1093/jxb/erm262

[B24] Kempers R. Ammerlaan A. van Bel A. J. E. (1998). Symplasmic constriction and ultrastructural features of the sieve element/companion cell complex in the transport phloem of apoplasmically and symplasmically phloem-loading species. Plant Physiol. 116, 271–278

[B25] Knoblauch M. van Bel A. J. E. (1998). Sieve tubes in action. Plant Cell 10, 35–50

[B26] Knop C. Stadler R. Sauer N. Lohaus G. (2004). AmSUT1, a sucrose transporter in collection and transport phloem of the putative symplastic phloem loader *Alonsoa meridionalis*. Plant Physiol. 134, 204–214 14730068 10.1104/pp.103.029264 PMC316300

[B27] Kühn C. (2003). A comparison of the sucrose transporter systems of different plant species. Plant Biol. 5, 215–232

[B28] Kühn C. Hajirezaei M. R. Fernie A. R. Roessner-Tunali U. Czechowski T. Hirner B. (2003). The sucrose transporter StSUT1 localizes to sieve elements in potato tuber phloem and influences tuber physiology and development. Plant Physiol. 131, 102–113 12529519 10.1104/pp.011676 PMC166791

[B29] Kühn C. Grof C. P. (2010). Sucrose transporters of higher plants. Curr. Opin. Plant Biol. 13, 288–298 20303321 10.1016/j.pbi.2010.02.001

[B30] Lalonde S. Tegeder M. Throne-Holst M. Frommer W. Patrick J. W. (2003). Phloem loading and unloading of sugars and amino acids. Plant Cell Environ. 26, 37–56 16667512

[B31] Lichtner F. T. Spanswick R. M. (1981). Electrogenic sucrose transport in developing soybean cotyledons. Plant Physiol. 67, 869–874 16661771 10.1104/pp.67.4.869 PMC425789

[B32] Manning K. Davies C. Bowen H. C. White P. J. (2001). Functional characterization of two ripening-related sucrose transporters from grape berries. Ann. Bot. 87, 125–129

[B33] Minchin P. E. H. Thorpe M. R. (1984). Apoplastic phloem unloading in stem of bean. J. Exp. Bot. 35, 538–550

[B34] Minchin P. E. H. Thorpe M. R. (1987). Measurement of unloading and reloading of photo-assimilate within the stem of bean. J. Exp. Bot. 38, 211–220

[B35] Motulsky H. Christopoulos A. (2003). Fitting models to Biological Data Using Linear and Nonlinear Regression. San Diego, CA: GraphPad Software, Inc., 143–148

[B36] Neher E. (1992). Correction for liquid junction potentials in patch-clamp experiments. Methods Enzymol. 207, 123–130 1528115 10.1016/0076-6879(92)07008-c

[B37] Ng B. Barry H. (1995). The measurement of ionic conductivities and mobilities of certain less common organic ions needed for junction potential corrections in elelctrophysiology. J. Neurosci. Methods 56, 37–41 7715244 10.1016/0165-0270(94)00087-w

[B38] Nie P. Wang X. Hu L. Zhang H. Zhang J. Zhang Z. (2010). The predominance of the apoplasmic phloem-unloading pathway is interrupted by a symplasmic pathway during chinese jujube fruit development. Plant Cell Physiol. 51, 1007–1018 20400534 10.1093/pcp/pcq054

[B39] Oparka K. J. Duckett C. M. Prior D. A. M. Fisher D. B. (1994). Real-time imaging of phloem unloading in the root tip of *Arabidopsis*. Plant J. 6, 759–766

[B40] Patrick J. W. (2013). Fundamentals of phloem transport physiology, in Phloem: Molecular Cell Biology, Systemic Communication, Biotic Interactions, eds Thompson G. A. van Bel A. J. E. (Hoboken, NJ: Wiley-Blackwell), 30–59

[B42] Patrick J. W. Offler C. E. (1996). Post-sieve element transport of photoassimilates in sink regions. J. Exp. Bot. 47, 1165–117 21245245 10.1093/jxb/47.Special_Issue.1165

[B41] Patrick J. W. Turvey P. M. (1981). The pathway of radial transfer of photosynthate in decapitated stems of *Phaseolus vulgaris* L. Ann. Bot. 47, 611–621

[B43] Reinders A. Schulze W. Thaminy S. Stagljar I. Frommer W. B. Ward J. M. (2002). Intra- and intermolecular interactions in sucrose transporters at the plasma membrane detected by the split-ubiquitin system and functional assays. Structure 10, 763–772 10.1016/S0969-2126(02)00773-6 12057192

[B44] Reinders A. Sivitz A. B. Starker C. G. Gantt J. S. Ward J. M. (2008). Functional analysis of LjSUT4, a vacuolar sucrose transporter from *Lotus japonicus*. Plant Mol. Biol. 68, 289–299 10.1007/s11103-008-9370-0 18618272

[B45] Reinhold L. Kaplan A. (1984). Membrane transport of sugars and amino acids. Annu. Rev. Plant Physiol. 35, 45–83 10.1146/annurev.pp.35.060184.000401 PMC1091924 16660343

[B46] Rhodes J. D. Thain J. F. Wildon D. C. (1996). The pathway for systemic electrical signal conduction in the wounded tomato plant. Planta 200, 50–57 10.1007/BF00196648

[B47] Riesmeier J. W. Hirner B. Frommer W. B. (1993). Potato sucrose transporter expression in minor veins indicates a role in phloem loading. Plant Cell 5, 1591–1598 8312741 10.1105/tpc.5.11.1591 PMC160388

[B48] Ritchie R. J. Prvan T. (1996). Current statistical methods for estimating the *K*_*m*_ and V_max_ of Michaelis-Menten kinetics. Biochem. Edu. 24, 196–206 10.1016/S0307-4412(96)00089-1

[B49] Ruan Y.-L. Patrick J. W. (1995). The cellular pathways of post-phloem sugar transport in developing tomato fruit. Planta 196, 434–444 10.1007/BF00203641

[B50] Sauer N. (2007). Molecular physiology of higher plant sucrose transporters. FEBS Lett. 581, 2309–2317 10.1016/j.febslet.2007.03.048 17434165

[B51] Schneider S. Beyhl D. Hedrich R. Sauer N. (2008). Functional and physiological characterization of *Arabidopsis INOSITOL TRANSPORTER1*, a novel tonoplast-localized transporter for *myo*-Inositol. Plant Cell 20, 1073–1087 10.1105/tpc.107.055632 18441213 PMC2390729

[B52] Schulz A. Beyhl D. Marten I. Wormit A. Neuhaus E. Poschet G. (2011). Proton-driven sucrose symport and antiport are provided by the vacuolar transporters SUC4 and TMT1/2. Plant J. 68, 129–136 10.1111/j.1365-313X.2011.04672.x 21668536

[B53] Schulze W. Weise A. Frommer W. B. Ward J. M. (2000). Function of the cytosolic N-terminus of sucrose transporter AtSUT2 in substrate affinity. FEBS Lett. 485, 189–194 10.1016/S0014-5793(00)02180-3 11094165

[B55] Tegeder M. Ruan Y.-L. Patrick J. W. (2013). Roles of plasma membrane transporters in phloem function, in Phloem: Molecular Cell Biology, Systemic Communication, Biotic Interactions, eds Thompson G. A. van Bel A. J. E (Hoboken, NJ: Wiley-Blackwell), 63–101

[B56] Thomson A. B. R. (1977). Limitations of the Eadie-Hofstee plot to estimate kinetic parameters of intestinal transport in the presence of an unstirred water layer. J. Membr. Biol. 47, 39–57 458847 10.1007/BF01869046

[B57] Thomson A. B. R. Dietschy J. M. (1977). Derivation of the equations that describe the effects of unstirred water layers on the kinetic parameters of active transport processes in the intestine. J. Theor. Biol. 64, 277–294 10.1016/0022-5193(77)90357-5 557154

[B58] van Bel A. J. E. (1996). Interaction between sieve element and companion cell and the consequences for photoassimilate distribution. Two structural hardware frames with associated software packages in dicotyledons? J. Exp. Bot. 47, 1129–1140 10.1093/jxb/47.Special_Issue.1129 21245242

[B59] van Bel A. J. E. (2003a). The phloem, a miracle of ingenuity. Plant Cell Environ. 26, 125–150

[B60] van Bel A. J. E. (2003b). Transport phloem: low profile, high impact. Plant Physiol. 131, 1509–1510 12692310 PMC1540299

[B61] van Bel A. J. E. Koops A. (1985). Uptake of ^14^C-sucrose in isolated minor vein networks of *Commelina benghalensis* L. Planta 164, 362–369 10.1007/BF00402947 24249605

[B62] van Bel A. J. E. van Rijen H. V. M. (1994). Microelectrode-recorded development of the symplasmic autonomy of the sieve element/companion cell complex in the stem phloem of *Lupinus luteus* L. Planta 192, 165–175 10.1007/BF01089031

[B63] van Bel A. J. E. Hafke J. B. (2005). Physiochemical determinants of phloem transport, in Vascular Transport in Plants, eds Holbrook N. M. Zwieniecki M. (Amsterdam: Elsevier), 19–44 10.1016/B978-012088457-5/50004-6

[B64] van der Schoot C. van Bel A. J. E. (1989). Glass microelectrode measurements of sieve tube membrane potentials in internode discs and petiole strips of tomato (*Solanum lycopersicum* L.). Protoplasma 149, 144–154 10.1007/BF01322986

[B65] Viola R. Roberts A. G. Haupt S. Gazzani S. Hancock R. D. Marmiroli N. (2001). Tuberization in potato involves a switch from apoplastic to symplastic phloem unloading. Plant Cell 13, 385–398 11226192 10.1105/tpc.13.2.385 PMC102249

[B66] Voitsekhovskaja O. V. Pakhomova M. V. Syutkina A. V. Gamalei Y. V. Heber U. (2000). Compartmentation of assimilate fluxes in leaves. II. Apoplastic sugar levels in leaves of plants with different companion cell types. Plant Biol. 2, 107–112 10.1055/s-2000-9459

[B67] Walker N. A. Patrick J. W. Zhang W.-H. Fieuw S. (1995). Efflux of photosynthate and acid from developing seed coats of *Phaseolus vulgaris* L.: a chemisosmotic analysis of pump-driven efflux. J. Exp. Bot. 46, 539–549 10948232

[B68] Weber H. Borisjuk L. Heim U. Sauer N. Wobus U. (1997). A role for sugar transporters during seed development: molecular characterization of a hexose and a sucrose carrier in fava bean seeds. Plant Cell 9, 895–908 10.1105/tpc.9.6.895 9212465 PMC156966

[B69] Weise A. Barker L. Kühn C. Lalonde S. Buschmann H. Frommer W. B. (2000). A new subfamily of sucrose transporters, SUT4, with low affinity/high capacitance localized in enucleate sieve elements of plants. Plant Cell 12, 1345–1355 10948254 10.1105/tpc.12.8.1345 PMC149107

[B70] Weschke W. Panitz R. Sauer N. Wang Q. Neubohn B. Weber H. (2000). Sucrose transport into barley seeds: molecular characterization of two transporters and implications for seed development and starch accumulation. Plant J. 21, 455–467 10.1046/j.1365-313x.2000.00695.x 10758497

[B71] Winne D. (1977). Correction of the apparent Michaelis constant, biased by an unstirred layer, if a passive transport component is present. Biochim. Biophys. Acta 464,118–126 10.1016/0005-2736(77)90375-3 831787

[B72] Wright J. P. Fisher D. B. (1981). Measurement of sieve-tube membrane potential. Plant Physiol. 65, 1133–1135 10.1104/pp.65.6.1133 16661766 PMC425784

[B73] Wu G. L. Zhang X. Y. Zhang L. Y. Pan Q. H. Shen Y. Y. Zhang D. P. (2004). Phloem unloading in developing walnut fruit is symplasmic in the seed pericarp and apoplasmic in the fleshy pericarp. Plant Cell Physiol. 45, 1461–1470 10.1093/pcp/pch169 15564530

[B74] Zhang L. Y. Peng Y. B. Pelleschi-Travier S. Fan Y. Lu Y. F. Lu Y. M. (2004). Evidence for apoplasmic phloem unloading in developing apple fruit. Plant Physiol. 135, 574–586 10.1104/pp.103.036632 15122035 PMC429418

[B75] Zhang X. Y. Wang X. L. Wang X. F. Xia G. H. Pan Q. H. Fan R. C. (2006). A shift of phloem unloading from symplasmic to apoplasmic pathway is involved in developmental onset of ripening in grape berry. Plant Physiol. 142, 220–232 10.1104/pp.106.081430 16861573 PMC1557625

[B76] Zhou Y. Qu H. Dibley K. E. Offler C. E. Patrick J. W. (2007). A suite of sucrose transporters expressed in coats of developing legume seeds includes novel pH-independent facilitators. Plant J. 49, 750–764 10.1111/j.1365-313X.2006.03000.x 17253986

